# Retrospective comparison of three minimally invasive approaches for adrenal tumors: perioperative outcomes of transperitoneal laparoscopic, retroperitoneal laparoscopic and robot-assisted laparoscopic adrenalectomy

**DOI:** 10.1186/s12894-020-00637-y

**Published:** 2020-06-09

**Authors:** Changwei Ji, Qun Lu, Wei Chen, Feifei Zhang, Hao Ji, Shiwei Zhang, Xiaozhi Zhao, Xiaogong Li, Gutian Zhang, Hongqian Guo

**Affiliations:** Department of Urology, Drum Tower Hospital, Medical School of Nanjing University, Institute of Urology, Nanjing University, 321 Zhongshan Rd, Nanjing, 210008 Jiangsu People’s Republic of China

**Keywords:** Adrenal tumor, Adrenalectomy, Laparoscopy, Robot-assisted surgery, Retroperitoneal

## Abstract

**Background:**

To compare the perioperative outcomes of transperitoneal laparoscopic (TLA), retroperitoneal laparoscopic (RLA), and robot-assisted transperitoneal laparoscopic (RATLA) adrenalectomy for adrenal tumors in our center.

**Methods:**

Between April 2012 and February 2018, 241 minimally invasive adrenalectomies were performed. Cases were categorized based on the minimally invasive adrenalectomy technique. Demographic characteristics, perioperative information and pathological data were retrospectively collected and analyzed.

**Results:**

This study included 37 TLA, 117 RLA, and 87 RATLA procedures. Any two groups had comparable age, ASA score, Charlson Comorbidity Index, and preoperative hemoglobin. The tumor size for RLA patients was 2.7 ± 1.1 cm, which was significantly smaller compared to patients who underwent TLA/RATLA (*p* = 0.000/0.000). Operative time was similar in any two groups, while estimated blood loss was lower for RATLA group (75.6 ± 95.6 ml) compared with the TLA group (131.1 ± 204.5 ml) (*p* = 0.041). Conversion to an open procedure occurred in only one (2.7%) patient in the TLA group for significant adhesion and hemorrhage. There were no significant differences between groups in terms of transfusion rate and complication rate. Length of stay was shorter for the RATLA group versus the TLA/RLA group (*p* = 0.000/0.029). In all groups, adrenocortical adenoma and pheochromocytoma were the most frequent histotypes.

**Conclusions:**

Minimally invasive adrenalectomy is associated with expected excellent outcomes. In our study, the RATLA approach appears to provide the benefits of decreased estimated blood loss and length of stay. Robotic adrenalectomy appears to be a safe and effective alternative to conventional laparoscopic adrenalectomy.

## Background

The incidence of adrenal tumors in the population is approximately 1.5 to 9.0% [1]. About 10% of adrenal tumors are functional, and less than 5% are malignant. Laparoscopic adrenalectomy was first described in 1992 [2]. At present it has routinely replaced the open procedure as the standard treatment for adrenal tumors, with the advantages such as less hemorrhage, shorter hospital stay, and minimally invasive incision [3–5]. Conventional laparoscopic adrenalectomy is generally performed by a transperitoneal laparoscopic (TLA) or retroperitoneal laparoscopic (RLA) approach. TLA is suitable for all sizes of tumors because of the large operating space, while RLA is a more direct approach to the adrenal gland, eliminating the dissection of adjacent structures. Nevertheless, laparoscopy is recognized as associated with a steep and prolonged learning curve for the nonarticulated instruments and inefficient force transmission.

After the application of the da Vinci Surgical System, robotic adrenalectomy has been increasingly performed instead of laparoscopic approach, showing the superior characteristics of magnified three-dimensional imaging and fully articulating instruments [6, 7]. Moreover, the procedure has been often criticized for the longer operation time and obviously higher cost compared with laparoscopic approach [8].

The aim of the present study was to compare the perioperative outcomes of robot-assisted TLA (RATLA) and conventional laparoscopic adrenalectomy (TLA and RLA) in a high-volume center. The study was presented at the 34th Annual EAU Congress [9].

## Methods

### Patients

This was a retrospective comparative study and consecutive cases of minimally invasive adrenalectomy performed between April 2012 and February 2018 were collected. The operative approaches performed included TLA, RLA, and RATLA. Finally, 241 patients were selected in this study: 37 (15.4%) patients underwent TLA, 117 (48.5%) underwent RLA, and 87 (36.1%) underwent RATLA. All of the operations were performed by one surgeon (Hongqian Guo). The American Society of Anesthesiologists (ASA) and the Charlson Comorbidity Index (CCI) were categorized for all patients according to the grading criteria [10, 11]. Complications were classified by the modified Clavien-Dindo classification system, which consisted of 5 severity grades [12]. We arranged an endocrinological consultation for each patient preoperatively to identify functional tumors. Patients who had functional adrenal lesions were treated for metabolic abnormalities and hypertension preoperatively. Preoperative imaging examinations included a CT scan or MRI for adrenal.

### Surgical technique

The surgical approach was selected according to the surgeon’s preference, physical condition of patient and the features of adrenal tumors. The specific surgical techniques for laparoscopic adrenalectomy and RATLA are described in detail previously [13–15]. The pictures of our robotic surgical team during surgery were presented in Additional file 1. For TLA, the patient was in the lateral position. The ports were routinely inserted at umbilicus and subcostal area in the midclavicular and axillary front lines. For RLA, the first incision was cut below the twelfth rib tip, and the surgeon dissected the retroperitoneal space directly with middle finger. Then, the next two ports were inserted under the direct vision of laparoscopy in midaxillary and axillary front lines. RATLA involved the use of the Si da Vinci robot. The patient was placed in lateral position at about 30 to 60 degrees. There were three robotic trocars, one 12-mm port for the 30-degree downward-angled camera and two 7-mm ports for the da Vinci tools, with monopolar scissors on the right arm and Fenestrated or Maryland grasper on the left arm. The two arms should be kept 8 cm away from the camera port to avoid obstruction. In addition, if needed, one 12-mm assistance port or one 5-mm port to recline the liver were included.

### Statistical analysis

Continuous variables are presented as the mean ± standard deviation. Student *t*-test (for data with normal distribution) was used to compare continuous variables, and Pearson x^2^ or Fisher exact test was used to compare categorical variables. Univariable and multivariable regressions were used to identify factors predicting estimated blood loss. All reported *p* values were 2-sided, and p value < 0.05 was considered statistically significant. Statistical analysis was performed using SPSS 17.0 (SPSS Inc., Chicago, IL).

## Results

Overall, 37 TLA, 117 RLA, and 87 RATLA patients who underwent procedures were analyzed. Demographic characteristics, perioperative data and pathological results are reported in Table 1. Any two groups had comparable age, ASA score, Charlson Comorbidity Index, and preoperative hemoglobin. Body mass index (BMI) was high in RLA group compared with the TLA group (*p* = 0.034). The tumor size for RLA patients was 2.7 ± 1.1 cm, which was significantly smaller compared to patients who underwent TLA/RATLA (*p* = 0.000/0.000). Of all patients, we found 95 nonfunctional and 146 functional adrenal tumors. Among the functional tumors, there were 58 aldosteronomas, 53 pheochromocytomas, and 35 hypercortisolism.

**Table 1:** Demographic and clinical parameters in the study

Parameters	Transperitoneal LA (TLA), *n* = 37	Retroperitoneal LA (RLA), *n* = 117	RATLA, *n* = 87	p^a^	p^b^	p^c^
**Age, yr, mean (SD)**	47.6 ± 12.1	49.3 ± 12.1	48.4 ± 14.0	0.447	0.746	0.638
**Gender**				0.231	0.844	0.188
**Male**	19 (51.4)	47 (40.2)	43 (49.4)			
**Female**	18 (48.6)	70 (59.8)	44 (50.6)			
**BMI, mean (SD)**	23.6 ± 2.9	24.9 ± 3.1	24.7 ± 3.9	**0.030**	0.081	0.809
**ASA score ≥ 3**	25 (67.6)	75 (64.1)	62 (71.3)	0.700	0.681	0.281
**Charlson Comorbidity Index ≥ 4**	6 (16.2)	14 (12.0)	19 (21.8)	0.697	0.475	0.058
**Side**				0.455	0.556	0.858
**Left**	17 (45.9)	62 (53.0)	45 (51.7)			
**Right**	20 (54.1)	55 (47.0)	42 (48.3)			
**Tumor size, mm, mean (SD)**	5.2 ± 2.1	2.7 ± 1.1	4.7 ± 2.6	**0.000**	0.253	**0.000**
**Past abdominal surgery**	5 (13.5)	16 (13.7)	14 (16.1)	0.980	0.715	0.630
**Preop Hb, g/dl, mean (SD)**	130.8 ± 18.6	133.4 ± 15.5	135.0 ± 16.7	0.400	0.209	0.458
**Operative time, min, mean (SD)**	141.6 ± 47.5	129.8 ± 38.8	136.1 ± 41.5	0.130	0.521	0.268
**Estimated blood loss, ml, mean (SD)**	131.1 ± 204.5	85.4 ± 88.8	75.6 ± 95.6	0.194	**0.041**	0.452
**Open conversion**	1 (2.7)	0 (0.0)	0 (0.0)	0.542	0.658	–
**Intraoperative transfusions**	2 (5.4)	2 (1.7)	3 (3.4)	0.523	0.994	0.736
**Postoperative transfusions**	3 (8.1)	2 (1.7)	1 (1.1)	0.167	0.147	1.000
**Complications**						
**Intraoperative**	3 (8.1)	5 (4.3)	3 (3.4)	0.623	0.516	1.000
**Postoperative**	4 (10.8)	14 (12.0)	6 (6.9)	1.000	0.710	0.229
**Clavien 1–2**	4 (10.8)	13 (11.1)	6 (6.9)			
**Clavien 3–4**	0 (0.0)	1 (0.9)	0 (0.0)			
**Hb at discharge, g/dl, mean (SD)**	116.3 ± 19.4	121.7 ± 15.5	121.2 ± 16.2	0.088	0.150	0.840
**Length of stay, d, mean (SD)**	6.6 ± 2.4	5.0 ± 3.8	4.1 ± 1.9	**0.024**	**0.000**	**0.029**
**Readmission**	0 (0.0)	2 (1.7)	2 (2.3)	1.000	0.880	1.000
**Tumor histology**				1.000	1.000	0.736
**Malignant**	1 (2.7)	2 (1.7)	3 (3.4)			
**Benign**	36 (97.3)	115 (98.3)	84 (96.6)			
**Histology type**				0.307	0.664	0.588
**Adrenocortical adenoma**	16 (43.2)	64 (54.7)	40 (46.0)			
**Pheochromocytoma**	6 (16.2)	27 (23.1)	18 (20.7)			
**Myelolipoma**	4 (10.8)	9 (7.7)	13 (14.9)			
**Adrenocortical carcinoma**	1 (2.7)	1 (0.7)	1 (1.1)			
**Metastasis**	0 (1.0)	1 (0.7)	1 (1.1)			
**Others**	10 (27.0)	15 (12.8)	14 (16.1)			

Data are n (%) unless indicated

^a^ Transperitoneal LA vs Retroperitoneal LA

^b^ Transperitoneal LA vs RATLA

^c^ Retroperitoneal LA vs RATLA

Operative time was similar in any two groups, while estimated blood loss was lower for RATLA group (75.6 ± 95.6 ml) compared with the TLA group (131.1 ± 204.5 ml) (*p* = 0.041). Conversion to an open procedure occurred in only one (2.7%) patient in the TLA group for significant adhesion and hemorrhage. There were no significant differences between groups in terms of transfusion rate and complication rate. The rate of intraoperative complication was low in all three groups, and most patients who had intraoperative complication developed an episode of hypertension or hemorrhage. The postoperative complication mainly consisted of grade 1 and 2 complications, with only one patient in RLA experiencing grade 3 complication. Length of stay was shorter for the RATLA group compared with the TLA/RLA group (*p* = 0.000/0.029), and shorter for the RLA group compared with the TLA group (*p* = 0.024). The total treatment costs added up to ¥27,696 for TLA, ¥27,138 for RLA and ¥68,150 for RATLA procedures. In all groups, adrenocortical adenoma and pheochromocytoma were the most frequent histotypes. At a median follow-up of 36 months (range 15–68), no imaging recurrence was observed.

On univariate analysis, the TLA versus RATLA procedure (*p* = 0.041) affected estimated blood loss (Table 2). On multivariate analysis, the tumor size and type of procedure (TLA vs RATLA) were significant predictive factors for estimated blood loss. (Table 3).

**Table 2:** Univariate analysis of estimated blood loss

Variable	Estimated blood loss	*p* Value
**Age**		0.602
**≥ 50**	93.3 ± 108.7	
**< 50**	85.4 ± 124.5	
**Gender**		0.178
**Male**	100.4 ± 152.7	
**Female**	80.0 ± 75.2	
**BMI**	NA	0.078
**Preop Hb**	NA	0.122
**Surgical procedure**		
**TLA vs RATLA**	131.1 ± 204.5 vs 75.6 ± 95.6	**0.041**
**RLA vs RATLA**	85.4 ± 88.8 vs 75.6 ± 95.6	0.452

**Table 3:** Multivariate analysis of estimated blood loss

Variable	*p* Value
**Age**	0.631
**BMI**	0.137
**Tumor size**	**0.022**
**Preop Hb**	0.330
**TLA vs RATLA**	**0.039**
**RLA vs RATLA**	0.134

## Discussion

Recently laparoscopic adrenalectomy has been the standard surgical procedure for adrenal tumors gradually [16]. Except the acknowledged benefits of laparoscopic surgery, surgeons need to overcome some specific disadvantages, including hand fatigue, the inferior ergonomic instruments, and a two-dimensional surgical view. The robotic system can provide three-dimensional imaging, more precise operation with fewer complications, the elimination of hand tremor, and reduction of surgeon’s fatigue, leading to quick recovery of patient while providing feasible oncological and perioperative outcomes [17]. Moreover, robotic adrenalectomy may benefit from a shorter learning curve and intracorporeal sewing compared with laparoscopic approach [18]. In addition, robotic surgery may offer a better selection for obese patients or those with large tumors [19].

In the present study, we compared the perioperative outcomes of three minimally invasive adrenalectomy approaches, including RATLA, TLA, and RLA in a high-volume center. Only a few previous studies exist that compare the results for all these three widely used techniques for minimally invasive adrenalectomy [20, 21]. Although the potential advantages to the surgeon have been shown in robotic adrenalectomy, most studies have demonstrated that the robotic approach has non-inferior oncologic results and perioperative outcomes, when compared with the laparoscopic approach [22]. In the present study, the results suggested that RATLA is as safe and effective as laparoscopic approach, and robotic approach was superior to laparoscopic approach in regard to blood loss and length of hospital stay. Robotic adrenalectomy may also help with partial adrenalectomy by providing clearer visualization and maneuverability to preserving the normal adrenal cortex and venous drainage. Another aspect of disadvantage with robotic approach was the higher cost compared with conventional laparoscopic and open adrenalectomy [8].

Conversion to an open procedure occurred in only 1 patient in the TLA group for significant adhesion and hemorrhage. No Conversion occurred in the RLA/RATLA group. Regarding the complications, most studies have shown that the risk of perioperative complications in robotic surgery is similar to or lower than that of conventional laparoscopic surgery [6]. In this study, minimally invasive adrenalectomy was performed with excellent perioperative outcomes, regardless of the approach. Most complications were mild, mostly consisted of nausea, pneumonia, hypokalemia, hematoma, and blood transfusion. Only 1 patient in the RLA group experienced grade 3 complication of retroperitoneal hematoma requiring reoperation.

Tumor size in patients undergoing RLA was significantly smaller compared to patients who underwent TLA/RATLA. This difference reflected the influence of selection bias, that is, the patients with smaller tumors were more likely to accept retroperitoneal approach, which may explain the shorter length of stay for the RLA group compared with the TLA group. In any two groups the operative time was similar, while the previous study demonstrated longer operative time in the robotic group compared with the laparoscopic group [22]. During follow-up, no imaging recurrence was observed in all patients probably for the benign characteristics of adrenal tumors.

The management of adrenocortical carcinoma is still controversial [23–25]. In this study, adrenocortical carcinoma was resected to clear margins in 2 patients of laparoscopic approach and in 1 patient of robotic approach. Of the 3 patients, one has developed local recurrence at the adrenalectomy bed during follow up.

Most patients with adrenal tumors often showed an abnormal and complex endocrinological presentation. All patients in our study received a preoperative consultation of endocrinology to determine functional tumors. We always think that an multidisciplinary model of preoperative preparation, operation, and long-term follow-up is necessary [26], and we strongly suggest a sufficient discussion between urologists and endocrinologists to ensure the patient’s safety. Although perioperative blood pressure control and monitoring is strictly enforced, the results show that endocrinological events, such as hypotension and hypokalemia, account for the majority of intraoperative complications.

The limitations of this study include the retrospective nature of the analysis, the selection bias of patients, and the confounding influences of the inherent bias in surgical approach selection. Other important outcomes variables, including patient satisfaction, postoperative pain, and cost, were not evaluated in the current study. Finally, the study lacks a long-term follow-up.

## Conclusions

Our study revealed that robotic adrenalectomy is as safe and effective as laparoscopic approach. The RLA approach appears to provide the benefits of decreased length of stay compared with TLA. The RATLA approach show the benefits of decreased estimated blood loss and length of stay. Robotic adrenalectomy is a safe and effective alternative to conventional laparoscopic approach.

## Supplementary information

**Additional file 1.**

## Data Availability

The datasets used and analysed during the current study are available from the corresponding author on reasonable request.

## Abbreviations

TLA: Transperitoneal laparoscopic adrenalectomy
RLA: Retroperitoneal laparoscopic adrenalectomy
RATLA: Robot-assisted transperitoneal laparoscopic adrenalectomy
ASA: American Society of Anesthesiologists
CCI: Charlson Comorbidity Index
BMI: Body mass index
