# Supplementary figures and images for: Retrospective comparison of three minimally invasive approaches for adrenal tumors: perioperative outcomes of transperitoneal laparoscopic, retroperitoneal laparoscopic and robot-assisted laparoscopic adrenalectomy
Source: BMC Urol. 2020 Jun 9;20:66. doi: 10.1186/s12894-020-00637-y (PMC7285739; doi:10.1186/s12894-020-00637-y)

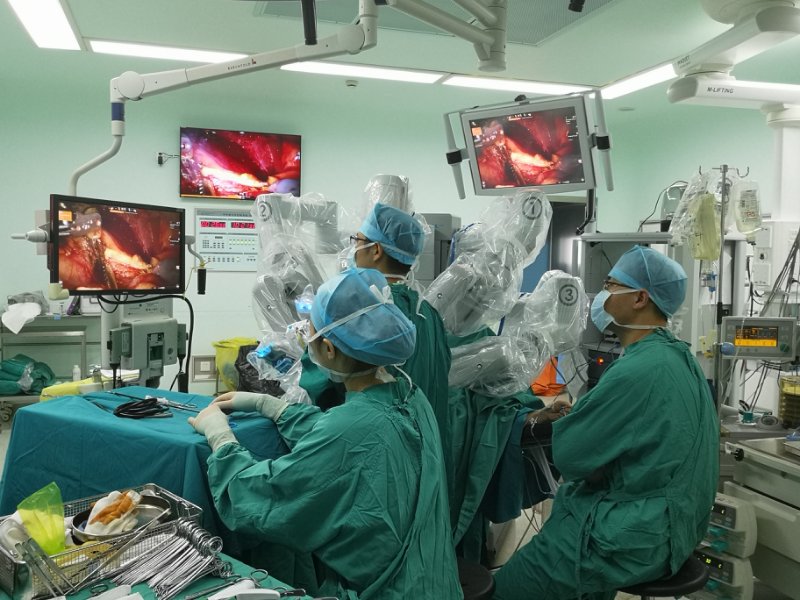

Supplement: Supplementary file 1 — Additional file 1. [file 12894_2020_637_MOESM1_ESM.jpg]
